# Revolutionization in Cancer Therapeutics via Targeting Major Immune Checkpoints PD-1, PD-L1 and CTLA-4

**DOI:** 10.3390/ph15030335

**Published:** 2022-03-09

**Authors:** Pratibha Pandey, Fahad Khan, Huda A. Qari, Tarun Kumar Upadhyay, Abdulhameed F. Alkhateeb, Mohammad Oves

**Affiliations:** 1 Department of Biotechnology, Noida Institute of Engineering and Technology, Greater Noida 201306, India; pratibhapandey.bio@niet.co.in; 2 Department of Biological Science, Faculty of Sciences, King Abdulaziz University, Jeddah 21589, Saudi Arabia; hagari@kau.edu.sa; 3 Animal Cell Culture and Immunobiochemistry Lab, Department of Biotechnology, Parul Institute of Applied Sciences and Centre of Research for Development, Parul University, Vadodara 391760, India; tarun_bioinfo@yahoo.co.in; 4 Department of Electrical & Computer Engineering, Faculty of Engineering, King Abdulaziz University, Jeddah 21589, Saudi Arabia; afalkhateeb@kau.edu.sa; 5 Center of Excellence in Environmental Studies, King Abdulaziz University, Jeddah 21589, Saudi Arabia

**Keywords:** cancer therapeutics, immunotherapy, immunogenic cell death, immune checkpoints, PD-L1/PD-1, CTLA-4

## Abstract

Numerous research reports have witnessed dramatic advancements in cancer therapeutic approaches through immunotherapy. Blocking immunological checkpoint pathways (mechanisms employed by malignant cells to disguise themselves as normal human body components) has emerged as a viable strategy for developing anticancer immunity. Through the development of effective immune checkpoint inhibitors (ICIs) in multiple carcinomas, advances in cancer immunity have expedited a major breakthrough in cancer therapy. Blocking a variety of ICIs, such as PD-1 (programmed cell death-1), programmed cell death-ligand 1 (PD-L1), and cytotoxic T-lymphocyte-associated protein 4 (CTLA-4) has improved the immune system’s efficacy in combating cancer cells. Recent studies also supported the fact that ICIs combined with other potent antitumor candidates, such as angiogenic agents, could be a solid promising chemopreventive therapeutic approach in improving the effectiveness of immune checkpoint inhibitors. Immune checkpoint blockade has aided antiangiogenesis by lowering vascular endothelial growth factor expression and alleviating hypoxia. Our review summarized recent advances and clinical improvements in immune checkpoint blocking tactics, including combinatorial treatment of immunogenic cell death (ICD) inducers with ICIs, which may aid future researchers in creating more effective cancer-fighting strategies.

## 1. Introduction

The recent era has witnessed enormous advancements in the oncology field by introducing the immunotherapy concept in cancer therapeutics. ICIs have emerged as one of the most potent therapeutic options in numerous solid tumors. Three immune checkpoint regulators, namely CTLA-4, PD-1, and PD-L1 have gained enormous attention in the oncology field as promising potent targets for cancer therapeutics [1]. In therapeutic approaches involving immune checkpoint inhibitor treatment, antibodies against the CTLA-4 protein, PD-1, or PD-L1 are employed [2]. As a result, ICIs regulate the interaction of cytotoxic T lymphocytes in the tumor microenvironment with tumor cells that have outlived their function. For instance, tumors recruit various suppressor immune cells, including myeloid-derived cells, Treg regulatory cells, and tumor-associated macrophages. Additionally, interactions between immune checkpoint receptors and ligands obstruct anti-tumor immunity [3]. Therefore, tumors express immune co-inhibitory ligands, including PD-L1, whereas tumor-infiltrating CTLs lymphocytes express the PD-L1 receptor, PD-1. Tumors also produce numerous immunosuppressive cytokines (TGF-β and IL-10), or reactive oxygen species (ROS) and nitric oxide to cease immune responses [4,5]. Immune checkpoint and co-inhibitory receptors have been recognized to play crucial roles in immune homeostasis maintenance. In fact, several co-inhibitory receptors have been genetically associated with autoimmune disorders, and have also been reported to be a key factor in regulating the control of immune responses. There are various mechanisms that are employed by cancer cells to circumvent the human immune system, including evasion of immune cells recognition, increased resistance to apoptotic pathways, or immunosuppressive conditions. Immune checkpoints have been considered as negative regulators of IR (immune response) in preventing excess peripheral tissue damage [6]. In 1997, the nuclear magnetic resonance (NMR) structure of human CTLA-4 (PDB: 1AH1), a receptor similar to PD-1 in function, was published, providing the first structural information on immune checkpoint proteins. Immune checkpoints (PD-L1 and PD-1) are membrane protein receptors with classical Ig (immunoglobulin-like) extracellular domains which engage with intracellular domains and transmit signals to them. Monocytes, T cells, natural killer T cells, B cells, and dendritic cells express CD279 (type I transmembrane receptor PD-1). PD-1 has two natural ligands: PD-L1 (B7-H1, CD274) and PD-L2 (B7-DC, CD273). Both proteins were found over a decade ago, and their roles in PD-1/PD-L1/2 signal transduction have been thoroughly investigated and are now well understood. Ig-like transmembrane receptors (PD-L1, PD-L2, and PD-1) are cell surface receptors. PD-L1 expression is constitutively reported in both non-hematological and hematopoietic cells and regulated by external stimuli. PD-L2 has been found to be expressed on the surface of mast cells, macrophages, specific B cell populations, and dendritic cells. The PD-1/PD-L1 system reduces T lymphocyte proliferation, cytokine production, and cytotoxicity in cancer cells, causing fatigue and apoptosis of tumor-specific T cells and allowing cancer cells to dodge immune response [7]. On the surface of various tumor types, including lymphoma, melanoma, lung and breast cancer, glioblastoma, ovarian, kidney, and bladder cancers, PD-L1 is frequently overexpressed. It is important to recognize the multiplicity of immunological functions that inhibitors of multiple immune checkpoints govern when thinking about their mode of action. For example, the two immune-checkpoint receptors, cytotoxic T-lymphocyte-associated antigen 4 (CTLA-4; also known as CD152) and programmed cell death protein 1 (PD-1; also known as CD279), which are both inhibitory receptors, regulate immune responses at different levels and through different mechanisms. Antibodies that block either of these receptors have clinical effects, implying that antitumor immunity can be improved on numerous levels and those combinatorial tactics can be rationally created using molecular considerations and preclinical models. The CTLA-4 receptor has only been found on T cells and regulates T cell activation in the early stages. CD28 has no effect on T cell activation unless the TCR is first touched by cognate antigen. CD28 signaling enhances TCR signaling after antigen recognition, causing T cells to become activated. CD28 and CTLA-4 ligands are identical (CD80 and CD86). Although the specific mechanisms of CTLA-4 activity are unknown, it has been postulated that its presence on the surface of T cells dampens T cell activation by actively conveying inhibitory signals to the T cell by outcompeting CD28 in binding CD80 and CD86. CTLA-4 inhibition improves a wide range of immunological responses that rely on helper T cells, while CTLA-4 interaction on TReg cells improves their suppressive activity. Treg cells produce CTLA-4 constitutively because it is a target gene of the forkhead transcription factor FOXP3, whose expression determines the Treg cell lineage. PD-1’s main function (in contrast to CTLA-4) is to inhibit T cell activation in peripheral tissues during an inflammatory response to infection and to prevent autoimmunity. Within the tumor microenvironment, this corresponds to a key immune resistance mechanism. When T cells become stimulated, PD-1 expression is induced. When one of its ligands binds to it, PD-1 suppresses kinases implicated in T cell activation via the phosphatase SHP250. However, other signaling pathways are likely to be triggered as well. Because PD-1 blocks the TCR’ ‘stop signal’, this pathway has the potential to alter the duration of T cell–APC or T cell–target cell contact. PD-1 is strongly expressed on Treg cells (similar to CTLA-4) and may increase their proliferation in the presence of a ligand. PD-1 has a broader expression than CTLA-4: it is induced on other activated non-T lymphocyte subsets, such as B cells and natural killer (NK) cells, limiting their lytic activity. Although PD-1 inhibition is commonly thought to increase the activity of effector T cells in tissues and the tumor microenvironment, it also increases the activity of NK cells in tumors and tissues and may increase antibody production either indirectly or directly through effects on PD-1+ B cells. Table 1 summarized several FDA-approved immune checkpoint blockade therapies against several tumor types. Upon checkpoint activation, the immune cell ceases to elicit a cytotoxic response. These checkpoint proteins have been regarded as crucial for the maintenance of balance between self-tolerance and autoimmunity [8].

**Figure 1:**
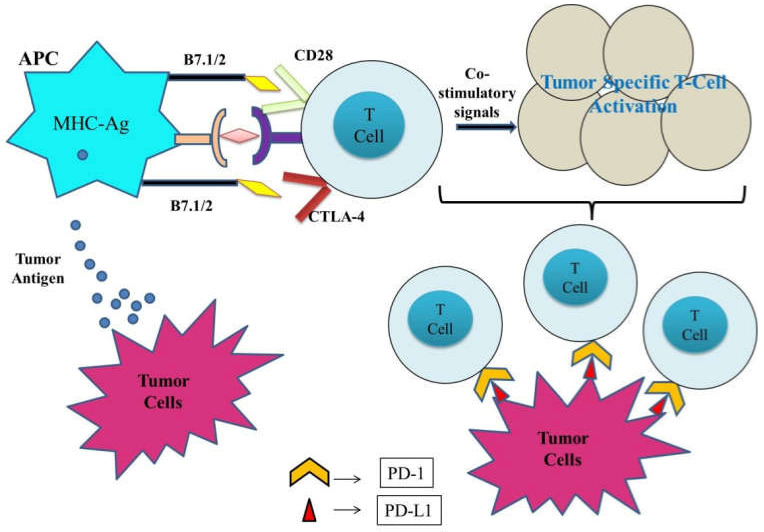
An overview of the correlation of several immune checkpoints with cancer initiation, progression, and development.

Targeting with PD-1, PD-L1, or CTLA-4 reverses the enervation of cytotoxic T lymphocytes, thereby leading to tumor cell elimination via the induction of the normal functioning of T cells. In immune-competent hosts, tumors need to escape immune surveillance for tumorigenesis. Blockade of PD-L1/PD-1 augments T cell growth, cytotoxicity, and tumor infiltration. Another critical T cell inhibitory receptor includes CTLA-4 which is expressed by Treg (regulatory T) cells and reported to be upregulated in activated T cells (Figure 1). In a number of studies, either in combination with other anti-PD-1 agents or on its own, anti-CTLA-4 has been shown to have anticancer properties [9].

## 2. Emerging Immune Checkpoint Inhibitors

### 2.1. CTLA-4 (CD152) Inhibitors

CTLA-4 is an immune checkpoint protein that is expressed on Treg cells and T-anergic cells. Human antibodies targeting CTLA-4 (cytotoxic T lymphocyte antigen-4) have displayed significant anticancer potential against multiple melanomas and tumor types [10]. It is a transmembrane protein whose extracellular surface receptor resembles the CD28 structure that facilitates competitive binding and also comprises a cytoplasmic domain having two tyrosine-based motifs required for signal transduction. It has also been involved in regulating the functioning of T cells and preventing immune cell-mediated damage. Upon TCR (T-cell receptor) activation, CTLA-4 gets trafficked into the cell membrane via TRIM (T-cell interacting molecules) and phosphorylated at Y201VKM, which remains attached to the cell surface [11]. Thereafter, CTLA-4 competes with CD28 for CD86 and CD80 ligands, preventing it from functioning as a costimulator for T-cell activation. CTLA-4 has also been reported to interact with GRB2, PI3K, filamin A, ZAP70, PKC, PTPN11, and PP2A (phosphatases), which are required for the initiation of an inhibitory response within T cells [12]. Dephosphorylated CTLA-4 permits the re-internalization of proteins into lysosomes and endosomes that can be further recycled upon T-cell activation. Several studies have reported its overexpression in several carcinomas, including breast cancer and esophageal cancer [13]. In 2011, ipilimumab was developed as the first ICI (immune checkpoint inhibitor) that gained FDA approval after its successful trials in metastatic melanomas. Ipilimumab is another human Ig (immunoglobulin) G1 mAb (monoclonal antibody) targeting CTLA-4 that sterically blocks the interaction of CTLA-4 with CD86 and CD80 on T cells or antigen-presenting cells to prevent the inhibitory potential of CTLA-4, thereby permitting the binding of CD28 to CD80/CD86 and leading to T cell activation. Ipilimumab has also been approved for its use in combination with nivolumab (PD-1 inhibitor) for the treatment of unresectable (advanced) melanoma, RCC, and MSI-h (metastatic microsatellite in-stability high) or dMMR (mismatch repair-deficient) colorectal cancer [14]. Another CTLA-4 inhibitor is Tremelimumab (human IgG2 mAb), having a similar course of action to ipilimumab, which blocks the interaction between CD28 and CTLA-4, thereby inhibiting CTLA-4 mediated inactivation of immune cells [15]. Tremelimumab has also been recognized as an orphan drug designation in malignant mesothelioma treatment showing modest clinical efficacy in phase IIb trial. However, in the additional phase IIb DETER-MINE trial, it has not attained trial endpoints during the third- or second-line treatment of unresectable malignant mesothelioma [16]. Tremelimumab in combination with several other immunomodulatory agents has also been examined in elucidating its clinical efficacies in other cancer types [17]. Further studies also displayed that CTLA-4 engagement activated intrinsic signaling cascades in T cells [18]. CTLA-4 activation has been reported with inhibition of IL-2 production and T cell proliferation and cell cycle phase arrest via cross-talks with numerous cell signaling pathways regulating cell proliferation, such as NFκB, PI3K, and MAPK signaling pathways [19,20,21]. Based on the significant anticancerous potential of CTLA-4 blockade in tumor models (murine), anti-CTLA-4 antibodies were promoted for development [22]. Amongst them, the ipilimumab inhibitor obtained approval for metastatic (unresectable) melanoma and surgery adjuvant for high-risk cancers (melanoma).

### 2.2. PD-1 (CD279) Inhibitors

PD-1 is a member of the CD28 family that has been reported to be expressed on various lymphoid cells, including T cells, NK (natural killer) cells, myeloid cells, and B cells [23]. It is a transmembrane protein having a single Ig (extracellular) variable domain and cytoplasmic domain comprising an immune receptor tyrosine-based on-switch and inhibitory motifs [24]. PD-L1 is a ligand for PD-1 and it transports co-inhibitory signal after binding with PD-L1 in the presence of the TCR signaling complex. This phenomenon is mediated by PD-1 phosphorylation and PTPN11 recruitment that dephosphorylates PCKθ, ZAP70, and CD3ζ, thereby limiting TCR/CD28 signaling [25]. Studies have also presented the inverse effect of PD-1 expression on the cytotoxic potential of natural killer cells, and cytokine production. In addition, PD-1 expression has been found to be reported to exhibit growth inhibitory potential against myeloid-derived cells viz. monocytes and macrophages [26]. Pembrolizumab and nivolumab are also clinically FDA-approved PD-1 inhibitors that have greatly improved the cure of several unresectable, advanced, and metastatic carcinomas [27]. Treatments with them have shown durable efficacies in a numerous range of malignancies. Currently, approximately more than 2000 clinical trials encompassing PD-1 inhibition are being sustained by NCI. Pembrolizumab and nivolumab are IgG4 antibodies that have high PD-1 binding efficacy and low affinity for Fc receptors and complements [28]. Both antibodies have a similar structure except for the difference in the Ag binding component of the antibody variable domain. Few studies have presented their similar efficacy in both in vitro and in vivo assays at a similar dose with limited side effects. Pembrolizumab has been recognized as the first ICI (immune checkpoint inhibitor) that has been approved for first-line treatment in several melanomas via inhibiting immune-cell suppression and deactivation [29]. It is also the first approved anticancer treatment in all carcinomas. Overall, several clinical studies have supported the significant anticancerous potential of pembrolizumab in patients having higher PD-L1 levels. Another FDA-approved PD-1 inhibitor is nivolumab which disrupts PD-1’s ability to negatively regulate T cell activation and proliferation via promoting binding of various PD-1 epitope to pembrolizumab. It has also been reported to be highly efficient against a wider range of carcinomas either in combination with ipilimumab or alone [30]. Nivolumab has also displayed durable responses in several pretreated and heavily mutated carcinomas, specifically in NSCLC, RCC, hepatocellular carcinoma, cHL, neck cancer, and melanoma [31]. Table 2 summarizes some potent emerging PD-1 inhibitors (reported in clinical trials) which have displayed significant PD-1 inhibition in preclinical trials.

### 2.3. PD-L1 (CD274) Inhibitors

PD-L1 (type 1 transmembrane protein) has been extensively expressed due to its involvement in attenuating immune response to infection and has been reported in numerous carcinomas, including ovarian cancer, melanoma, colon adenocarcinoma, lung squamous cell carcinoma, and breast adenocarcinoma [39]. PD-L1 expression can either be induced or constitutive. Constitutive PD-L1 expression has been correlated with oncogenic mutations, whereas induced PD-L1 expression can further be associated with inflammation via interferon-γ, TNF-α, interleukins (IL-1α and IL-1β) or TILs (tumor-infiltrating lymphocytes). In ER (endoplasmic reticulum) and Golgi apparatus, PD-L1 becomes glycosylated with N-linked glycoproteins before acting as a PD-1 ligand [40]. PD-L1 glycosylation results in its expression stabilization and increased resistance to degradation. The binding of PD-L1 and PD-1 leads to immunosuppression via inhibiting T cell activation and proliferation, cytokines release, and cell survival [41]. Both PD-L1 and PD-1 inhibitors have a similar mode of action of inhibition via sterically obstructing the interaction between immune checkpoint receptors and ligands.

Atezolizumab was also the first FDA-approved PD-L1 inhibitor (human IgG1 mAb) used to treat UC, TNBC, NSCLC, and SCLC (small cell lung cancer). It is a genetically engineered inhibitor with a modified Fc domain inhibiting ADCC (antibody-dependent cell-mediated cytotoxicity) engagement [42]. This Fc domain modification has been associated with preventing PD-L1 expression on body immune cells, thereby leading to effective anti-immune cell response. Avelumab is also the first FDA-approved human IgG1 mAb for Merkel cell carcinoma (metastatic) treatment, then later for RCC and UC treatment [43]. Durvalumab is another currently FDA-approved IgG1 anti-PD-L1 mAb that does not elicit ADCC (due to Fc modification) and is being used for the treatment of NSCLC and UC [12]. PD-L1 inhibition has been used as a feasible treatment strategy for NSCLC, UC, and Merkel cell carcinoma treatment. Other potent PD-L1 inhibitors include KN035 (anti-PD-L1 mAb) which is currently under phase 1 investigation in patients with solid tumors, and phase II investigation in HER2 +ve breast cancers and advanced solid tumors (NCT03667170), and biliary tract cancer (NCT03478488) [44]. Altogether, the atezolizumab drug is only effective in patients whose responses initially show a consequent speedy disease progression due to acquired and primary resistance to PD-L1/PD-1 inhibition. Further, studies are still being conducted to reverse or prevent resistance to several cancer therapies for better patient outcomes.

Adaptive and primary resistance to immunotherapy has been reported to be one factor that limits the therapeutic efficacy in patients undergoing immune checkpoint inhibitor treatment. This has urged the need to develop other methods to maximize the therapeutic effectiveness of ICI treatment by combining it with some other cancer therapies, including immunotherapy, radiotherapy, chemotherapy, or targeted therapies. These studies are not limited to enhancing the functioning and activation of immune cells associated with tumor cell destruction. Overcoming resistance mechanisms would be one of the critical factors needed to achieve enhanced efficacies of immunotherapy in cancers. Hence, future therapeutic strategies should not be limited to malignant cells but also their microenvironment and functioning of immune-competent cells. Future perspectives of immunotherapy in cancer management depend on patient access to these innovative treatments. Building on the accomplishments of immune checkpoint inhibitors, several immunotherapies have been explored to be used either in combination with some existing therapies or other immunotherapies (Table 3). For instance, anti-PD-1 has been reported in combination with oncolytic virus treatment, CAR T-cell therapy, cyclin-dependent kinase inhibitors.

## 3. Immune Checkpoint Inhibitors in Combination with ICDs (Immunogenic Cell Death) Inducers

Cancer immunotherapy is a promising treatment approach that focuses on strengthening the immune response against several cancers. However, effector T cell inhibition in the tumor immunosuppressive microenvironment and lower immunogenicity in tumor cells are two major hindrances in effective cancer management. ICD inducers have numerous anticancer potentials via increasing tumor immunogenicity and inducing an antitumor immune response in cancer cells. ICIs can further enhance immune cell inhibition and thus, combining ICD inducers with ICIs might elicit a significant antitumor efficacy against numerous carcinomas. ICD is characterized by DAMPs (damage damage-associated molecular patterns), the release of tumor antigens, and proinflammatory cytokines, which assist in the presentation and uptake of antigens to body immune cells, thereby stimulating a significant antigenic tumor specific immune response [65].

**Figure 2:**
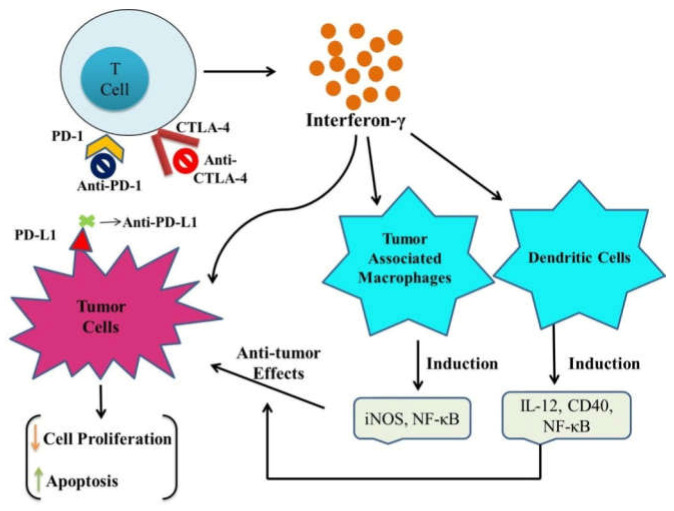
Possible molecular mechanism associated with main immune checkpoints (CTLA-4, PD-1, and PD-L1) blockage therapy in malignant cells. Immune checkpoint blockade activates the function of T cells that secrete IFN-γ which consecutively restores the antitumor potential of tumor-associated macrophages, and dendritic cells (Arrows indicates: ↑ Decreaseed and ↓ Increased).

Furthermore, effector T cell inhibition in tumor sites results in severe deterioration and metastasis. Immunotherapy involving the blockade of the immune checkpoint has shown remarkable efficacy in relieving immunosuppression and restoring the antitumor potential of immune cells, thereby demonstrating a practical cancer therapeutic approach. Commonly used immune checkpoints include PD-1/PD-L1, IDO, CTLA-4, and CD47. Figure 2 summarizes the possible molecular mechanism associated with blockage therapy in malignant cells with major immune checkpoints (CTLA-4, PD-1, and PD-L1). Thus, ICIs combined with ICD inducers could be a promising approach for enhanced antitumor effects. Studies reported by Kuai et al. strongly supported the antitumor efficacy of nanodiscs loaded with DOX (doxorubicin). Moreover, a combination of DOX-loaded nanodiscs and anti-PD-1 immunotherapy presented better antitumor immunity and sensitivity to IC blockade therapy in cancer patients. It sensitized tumors to the immune checkpoint blockade [66]. Table 4 summarizes the efficacy of combinatorial treatment of ICD inducers and immune checkpoint inhibitors.

Over the last few years, ICIs targeting PD-1, PD-L1, and CTLA-4 have drastically altered the therapeutic algorithm of numerous solid and hematological tumors. A critical challenge is presented by limited validated predictive biomarkers, which could help identify immunotherapy responders, an urgent need in numerous immunologically “cold” carcinomas where immune checkpoint inhibitors are still identifying their niche. Although monotherapies using CTLA-4-or PD-1-blocking antibodies helped some patients with specific malignancies live longer, the majority of patients did not respond. The discovery that combining ipilimumab and nivolumab resulted in more robust responses than either treatment alone cleared the door for the development of successful checkpoint therapy combinations, and numerous techniques are now being tested. Experiments in murine tumor models have shown that different checkpoint inhibitors can function in concert due to nonredundant processes [87]. This has been seen when anti-PD-1 is combined with anti-CTLA-4, anti-TIM-3, or anti-LAG-3. In a mouse colon carcinoma model, combining anti-PD-1 with an activating antibody against the costimulatory receptor 4-1BB improved antitumor responses.

An array of cancer antigens and adjuvants that activate innate immune cells, particularly DCs, is often included in cancer vaccines. Cancer vaccines are designed to create tumor-specific T lymphocytes that secrete IFN-γ or lytic granules, killing tumor cells. Immunological checkpoint inhibition combined with immune activators, such as cancer vaccines, may be especially beneficial for patients with malignancies that lack T cell infiltration. Animal models have provided some data in favor of this notion. According to Pérez-Ruiz et al., 2020, blocking PD-1 and CTLA-4 boosted the efficacy of a vaccine made up of irradiated Flt3 ligand-secreting B16 cells. In B16 tumors, a higher CD8/Treg ratio was linked to a decrease in tumor growth [88].

Furthermore, in a B16 cancer model, combining anti-CTLA-4 and anti-PD-1 inhibitors improved the efficacy of a vaccine for tumor lysates, GM-CSF and CpG. Compared to mice treated alone with the immunization, anti-PD-1 or anti-CTLA-4 dramatically increased the number of infiltrating IFN-γ secreting CD8+ T cells. In the CT26 model, PD-1 inhibition significantly improved the efficacy of a TLR-adjuvanted cancer vaccine and established robust anti-tumor memory, shielding mice from tumor growth upon rechallenge, according to a recent study. Increased effector T-cell responses and decreased Treg infiltration were linked to the protective effect. Immune checkpoint blockade has also been reported to boost the efficacy of non-immune therapies, such as radiotherapy and targeted cancer medicines. Combinations of immune checkpoint blockades and HDACi (histone deacetylase inhibitors) have shown auspicious results in treating peripheral or cutaneous T-cell lymphomas and malignant myeloma by inducing direct tumor cytotoxicity and increased tumor immunogenicity. Woods et al. also reported that HDACi stimulates PD-L1 expression in murine and human melanoma cells [89]. HDACi also increases the therapeutic efficacy of PD-1 inhibition via promoting the release of T-cell attractive chemokines into the tumor microenvironment [90]. In conjunction with targeting CTLA-4 and/or PD-1 and/or PD-L1, radiotherapy has been shown to induce CTL-mediated anti-tumor immunity in studies [91]. Combination therapy with PD-1 inhibition and brain-directed radiation, for example, resulted in anti-tumor effects with a 75% complete pathologic response and significantly improved OS in glioma xenograft-bearing mice due to CTL and macrophage activation.

Meanwhile, RT appeared to increase the M1/M2 ratio by stimulating macrophage repolarization. Other studies have found that RT combined with anti-PD-1 medication causes more severe lung injury in tumor-bearing animals and increased neutrophil infiltration and inflammatory response [92]. Recent studies have shown that increasing CXCL12 expression in HCC models alleviated hypoxia and increased the recruitment of immunosuppressive cells, whereas using a PD-1 inhibitor in combination with CXCR4 inhibition and sorafenib inhibited HCC growth [68]. The TIL population and activation in the glioma microenvironment were also sustained by dual targeting CXCR4 and PD-1. In glioma cell-bearing mice, targeting MDSCs with CXCR4 inhibition potentiated anti-PD-1 to maintain anticancer immune reactivity and improved OS [93].

## 4. Conclusions

ICI treatments have demonstrated enormous clinical benefits for a broader range of carcinomas. The addition of immune checkpoint inhibitors in the arsenal of immune therapeutics has paved a new way for researchers to identify better therapeutic approaches for the efficient management of cancer. Unrivaled responses have been reported amongst advanced staged cancer patients, such as lung cancer, melanoma, bladder cancer, and Hodgkin’s disease, treated with anti-CTLA-4, PD-1/L1 therapies, although a minimal percentage of patients benefitted after such treatments. Although much research has been done in recent years, the field of cancer immunotherapy has been going ahead at a faster pace. More research is still needed to be conducted to elucidate efficient immune checkpoint combinatorial approaches involving individual tumor genetics to predict better responses to these immunotherapy-based therapeutic approaches. Altogether, several combinatorial treatments incorporating immunotherapies and predictive biomarkers as cancer immunotherapeutic approaches might unravel a potent therapeutic approach for the better management of cancers.

## Figures and Tables

**Table 1:** List of PD-L1/PD-1 blockade approved therapies in different types of cancers.

Cancer	FDA Approved Agents	Clinical Trial (s)
Merkel cell carcinoma (Skin cancer)	Avelumab (2017)	JAVELIN phase 2
	Pembrolizumab (2018)	KEYNOTE-017 phase 2
Melanoma (Skin Cancer)	Pembrolizumab (2014)	KEYNOTE-001 phase 1
	Nivolumab (2014)	CheckMate-037
	Nivolumab + ipilimumab (2015)	CheckMate-069
	Pembrolizumab (2015)	KEYNOTE-006 phase 3
	Nivolumab + ipilimumab (2016)	CheckMate-067 phase 3
	Nivolumab (2017)	CheckMate-238 phase 3
Primary mediastinal large B-cell lymphoma (Blood cancer)	Pembrolizumab (2018)	KEYNOTE-170 phase 2
Classical Hodgkin lymphoma (Blood Cancer)	Nivolumab (2016)	CheckMate-039 phase 1 and Checkmate-205 phase 2
	Pembrolizumab (2017)	KEYNOTE-087 phase 2
Small cell lung cancer	Nivolumab (2018)	CheckMate-032 phase ½
Non-small cell lung cancer	Nivolumab (2015)	CheckMate-017 phase 3
	Pembrolizumab (2015)	CheckMate-057 phase 3
	Atezolizumab (2016)	POPLAR phase 2 and OAK phase 3
	Pembrolizumab (2016)	KEYNOTE-024 phase 3
	Pembrolizumab + Caroplatin + Pemetrexed (2017)	KEYNOTE-024 phase 3
	Durvalumab (2018)	KEYNOTE-021 phase 2
	Pembrolizumab + Pemetrexed + Platinum (2018)	PACIFIC phase 3 KEYNOTE-189 phase 3
Microsatellite instability-high and DNA mismatch repair deficiency unresectable solid tumors (Gastrointestinal Cancer)	Pembrolizumab (2017)	KEYNOTE-164 phase 2
	Nivolumab (2017)	CheckMate-142 phase 2
	Nivolumab + ipilimumab (2018)	CheckMate-142 phase 2
Hepatocellular carcinoma	Nivolumab (2017)	CheckMate-040 phase ½
	Pembrolizumab (2018)	KEYNOTE-224 phase 2
Gastric cancer	Pembrolizumab (2017)	KEYNOTE-059 phase 2
Renal cell cancer	Nivolumab (2015)	CheckMate-025 phase 3
	Nivolumab + ipilimumab (2018)	CheckMate-025 phase 3
Urothelial cancer (Renal Cancer)	Atezolizumab (2016)	IMVigor 210 phase 2
	Nivolumab (2017)	CheckMate-275 phase 2
	Atezolizumab (2017)	IMVigor 210 phase 2
	Durvalumab (2017)	Study 1108 phase 2
	Avelumab (2017)	JAVELIN solid tumor phase 1
	Pembrolizumab (2017)	KEYNOTE-052 phase 2 KEYNOTE-045 phase 3
Cervical cancer	Pembrolizumab (2018)	KEYNOTE-158 phase 2
Head and Neck squamous cell carcinoma	Pembrolizumab (2016)	KEYNOTE-012 phase 1b
	Nivolumab (2016)	CheckMate-141 phase 3

**Table 2:** FDA-approved PD-1 inhibitors which have displayed significant PD-1 inhibition in preclinical trials.

Emerging PD-1 Inhibitors	Features	Cancer Type	Reference
Cemiplimab	Fully human hinge stabilized IgG4 anti-PD-1 antibody	CSCC patients (both metastatic or locally advanced)	[32]
Sintilimab	PD-1 targeted human IgG4 mAb (monoclonal antibody)	Gastric carcinoma (NCT03745170)	[33]
		Lymphoma (NCT04052659)	
		Oesophageal carcinoma (NCT03946969)	
		NSCLC (NCT03830411)	
		Nasopharyngeal cancer (NCT03700476)	
Tislelizumab	PD-1 targeted humanized IgG4 mAb	Hodgkin’s lymphoma (both relapsed and refractory)	[34]
		Nasopharyngeal carcinoma (NCT03924986)	[35]
		UC (NCT03967977)	
		Gastroesophageal or gastric junction cancer (NCT03777657)	
		Lymphoma (NCT03493451)	
		Oesophageal carcinoma (NCT03957590)	
		NSCLC (NCT03358875)	
Toripalimab	Humanized IgG4 anti PD-1 mAb	Metastatic melanoma patients who did not respond to systemic therapies	[36]
		Liver cancer (NCT03949231)	[37]
		Oesophageal cancer (NCT03829969)	
		Neck and head cancer (NCT03952065),	
		Melanoma (NCT03941795),	
		NSCLC (NCT03924050),	
		Neuroendocrine carcinoma of the bladder (NCT03992911)	
		Nasopharyngeal carcinoma (NCT03581786)	
Spartaliumab	IgG4 PD-1 targeted mAb	Phase III COMBI-I trial (NCT02967692) in BRAFV600 mutant metastatic or unresectable melanoma	[38]
		triple-negative breast cancer treatment (TNBC; NCT03499899)	
		RCC (NCT04028245)	
		NSCLC (NCT03647488)	
		Nasopharyngeal carcinoma and colorectal cancer (NCT03891953)	

**Table 3:** Immune checkpoint inhibitors in cancer therapeutics.

Inhibitor	Role in Cancers	References
Atezolizumab (MPDL3280) Fully humanized IgG1 monoclonal antibody having a modified Fc domain that prohibits the depletion of PD-L1 expressing T cells	It blocks PD-L1 interaction with both B7.1 and PD-1.	[45]
	Atezolizumab treatment increased immunity against tumors via reducing immunosuppressive signals present in the tumor microenvironment.	[46]
	Highly effective against several hematologic malignancies and solid tumors.	[47]
	Several preclinical studies have reported increased CD8+ T, IL-18, CXCL11, IFN cells, and reduced IL-6 cytokines.	[48]
	In a phase 1 study, three dosing schedules of this drug were tested against various recurrent melanomas, renal cell carcinoma, non-small cell lung carcinoma, gastric cancer, and neck and head squamous cell cancer. In addition, phase II trials have reported a 10% overall response rate in patients with enhanced PD-L1 expression levels.	[49]
	Atezolizumab obtained FDA approval for metastatic or local advanced urothelial cancer against cisplatin therapy in May 2016.	[50]
	Commonly reported adverse effects include fatigue, pyrexia, reduced appetite, diarrhea, nausea, arthralgia, rash, pruritus, and headache.	
	Obtained FDA approval in October 2016 for NSCLC patients undergoing platinum-based chemotherapy	[51]
	In December 2018, atezolizumab obtained FDA approval for NSq NSCLC (non-squamous and non-small cell lung carcinoma) patients along with chemotherapy and bevacizumab treatments.	[52]
	In March 2019, it obtained further FDA approval for small cell lung cancer patients and chemotherapy. Further FDA has also granted its approval for metastatic or local advanced PD-L1 + ve TNBC patients along with nab-paclitaxel treatment.	[53]
Durvalumab (MEDI4736) Fully humanized IgG1 monoclonal antibody that blocks the interaction of CD80 molecules with PD-1.	Potent inhibitor with subnanomolar activities against PD-L1.	[54]
	In vivo studies having co implanted T cells have shown significant inhibition of human tumor growth in a xenograft model.	[55]
	In May 2017, FDA approved urothelial cancer patients (metastatic or locally advanced) following platinum-based chemotherapies.	[56]
	In February 2018, durvalumab received FDA approval for unresectable NSCLC (stage III) patients undergoing platinum-based chemotherapies.	[57]
Avelumab (MSB0010718C) Fully humanized IgG1 mAb blocking the interaction of PD-L1 with B7.1 and PD 1 (Inhibitory T cell receptor)	Avelumab treatments result in cytokine production or adaptive or cell-mediated antitumor IR (immune response).	[58]
	The wild-type Fc region helps the NK cells induce tumor-directed ADCC (antibody-dependent cell-mediated cytotoxicity).	[59]
	In March 2017, the FDA approved its use for patients with metastatic Merkel cell carcinoma (MCC).	[60]
	In May 2017, the FDA approved its usage for patients with metastatic urothelial cancer following platinum-based chemotherapy.	[61]
	In May 2019, the FDA approved its use for patients with advanced RCC (renal cell carcinoma) axitinib treatment.	[62]
BMS-936559 (MDX-1105) Fully humanized IgG4 mAb that inhibits binding of PD-L1with CD80 and PD-1	Commonly reported side effects include infusion reaction, arthralgia, fatigue, rash, headache, and pruritus.	[63]
	Studies showed an overall response rate of 17% in NSCLC, RCC, and melanoma patients.	
CK-301 Fully humanized IgG4 mAb of IgG1 that blocks the interaction of PD-L1 with B7.1 and PD-1	Comprises the functional Fc domain capable of ADCC induction and CDC (complement-dependent cytotoxicity) mediated Killing of PD-L1+ lymphoma cells.	[64]

**Table 4:** ICD inducers in combination with immune checkpoint inhibitors.

Cancer	ICD Inducers	ICIs	Anticancer Efficacy	Reference
Breast cancer	Doxil	IND (an IDO-1 inhibitor) and anti-PD-1 antibody	It induced superior synergistic anticancer response in comparison to DOX-only liposome along with reduced tumor volume	[67]
	Paclitaxel	NLG919 (an IDO-1 inhibitor)	Well-controlled tumor growth with a prolonged median survival time of mice	[68]
	OXA	NLG919 (an IDO-1 inhibitor)	High efficiency of combined drugs over tumor growth regression compared to free medicines and prevented metastasis in tumor-bearing mice.	[69]
	MIT	ND (an IDO-1 inhibitor)	Significant decrease in tumor size and increased survival rate in the treated animal.	[70]
	Doxil	NLG919 (an IDO-1 inhibitor)	Increased tumor growth inhibitory potential and prolonging survival rate in treated mice.	[71]
	Doxil	IND (an IDO-1 inhibitor)	Improved immune response and tumor regression in tumor-bearing mice	[72]
	Ce6 (Photosensitizer)	Anti-PD-1 antibody	Increased ROS production via PDT and elevated tumor ICD; evoked immune response	[73]
	Photosensitizer (pyrolipid, a lipid the conjugate of pyropheophorbidea)	Anti-PD-L1 antibody	Stimulated systemic immune response and distant tumors were inhibited	[74]
	ICG	Anti-PD-L1 antibody	Prevented liver and lung metastasis via activation of antitumor immune system	[75]
	Camptothecin + polypyrrole	Anti-PD-L1 antibody	Combined treatment-induced potent tumor immunogenic cell death and enhanced antitumor immune response. Prevented tumor recurrences and metastasis	[76]
	DOX + Ce6	Anti-PD-L1 antibody	Significant synergistic therapeutic effect was observed that eventually triggered the antitumor immune response and inhibited metastasis	[77]
	Ce6 + Magnetic hyperthermia	Anti-CTLA4 Antibody	Combinatorial treatment exhibited strong anticancer activity and elicited ICD along with eradication of metastatic tumors	[78]
Colon Cancer	DOX	Anti-PD-1 Antibody	Treatment resulted in complete regression of persisted tumors in animals and inhibited tumor recurrence in survivors	[67]
	Ce6	Anti-CTLA4 Antibody	This resulted in ICD induction and inhibition of distant tumors	[79]
	DOX + photothermal reagent	Anti-PD-L1 antibody	Treatment resulted in tumor cell death and induced effective ICD. In addition, it prevented tumor growth with stimulated immune response	[80]
	OXA + photosensitizer	Anti-PD-L1 antibody	Treatment resulted in tumor cell death and provoked ICD resulting in tumor regression via a strong immune response.	[81]
	OXA + DHA	Anti-PD-L1 antibody	Treatment retarded tumor growth initially for one month, and no tumor recurrence was reported for about 120 days	[82]
	OXA + PPa	Anti-CD47 Antibody	The treatment potentially inhibited tumor (both primary and abscopal) growth and inhibited tumor recurrence and metastasis	[83]
Prostate Cancer	Radiotherapy	Anti-PD-L1 antibody	Enhanced tumor ICD and tumor growth suppression, resulting in synergistic anticancer immune response	[84]
	IRE	IDO-1 inhibitor	Induced tumor ICD and overturned tumor immunosuppression, leading to the elimination of both secondary and primary tumors.	[85]
B-cell Lymphoma	DOX	IDO-1 inhibitor	Significant improvement in antitumor response in comparison to Doxil	[86]

## Data Availability

Not applicable.

## Abbreviations

PD-1/PD-L1	programmed death 1/programmed death ligand 1
CTLA-4	cytotoxic T lymphocyte-associated antigen-4
IDO	indoleamine 2.3-dioxygenase
HER2+	human epidermal growth factor receptor 2
SCLC	small cell lung cancer
TNF-α	tumor necrosis factor-α
CSCC	cutaneous squamous cell carcinoma
NCI	National Cancer Institute
FDA	Food and Drug Administration
CTLs	cytotoxic T lymphocytes
CTLA-4	cytotoxic T lymphocyte-associated protein 4
ROS	reactive oxygen species
IR	immune response
TCR	T cell receptor
TRIM	T cell interacting molecules
